# Measurements of source emittance and beam coherence properties of the upgraded Advanced Photon Source

**DOI:** 10.1107/S160057752500579X

**Published:** 2025-08-15

**Authors:** Xianbo Shi, Yu-Chung Lin, Jiyong Zhao, Thomas Toellner, Michael Y. Hu, Soenke Seifert, Byeongdu Lee, Walan Grizolli, Michael J. Wojcik, Luca Rebuffi, Lahsen Assoufid, Vadim Sajaev

**Affiliations:** a Advanced Photon Source Argonne National Laboratory 9700 S. Cass Avenue Lemont IL60439 USA; b Advanced Light Source Lawrence Berkeley National Laboratory 1 Cyclotron Road Berkeley CA94720 USA; Tohoku University, Japan

**Keywords:** source emittance, grating interferometry, transverse coherence, X-ray optics, Advanced Photon Source

## Abstract

The Advanced Photon Source (APS) has been upgraded with a multi-bend achromat lattice, achieving significantly reduced electron beam emittance and enhanced X-ray coherence. Precise characterization of these properties is essential for optimizing beamline performance and enabling new experiments that take full advantage of the upgraded source. We report on measurements of source size and transverse coherence properties using grating interferometry at two beamlines: the APS 3-ID-B undulator beamline and the 1-BM-B bending magnet beamline. The results confirm the world-record horizontal emittance of the upgraded APS below 30 pm rad and validate the theoretical design parameters. We further investigate the impact of optical aberrations and mechanical vibrations at the 12-ID-C beamline on coherence preservation. These measurements establish a benchmark for future beamline enhancements and demonstrate the effectiveness of grating interferometry for high-precision beam characterization. Our findings provide critical insights into synchrotron beam dynamics, coherence degradation mechanisms, and strategies for optimizing beamline X-ray optics at next-generation light sources.

## Abstract 2

This study presents source emittance and beam coherence measurements at the upgraded Advanced Photon Source using grating interferometry. The results confirm world-record emittance values, validate design parameters, and offer key insights into coherence preservation and beamline optimization.

## 1. Introduction

The Advanced Photon Source (APS) at Argonne National Laboratory has undergone a major upgrade, transitioning to a synchrotron radiation source based on a multi-bend achromat (MBA) lattice (Borland *et al.*, 2018 ▸). This upgrade significantly reduces the electron beam emittance, resulting in a two-order-of-magnitude increase in X-ray source brightness and coherent flux. These advances open new avenues for advanced X-ray techniques, such as coherent diffraction imaging (CDI) (Robinson *et al.*, 1999 ▸; Prosekov *et al.*, 2021 ▸; Chushkin & Zontone, 2025 ▸), X-ray photon correlation spectroscopy (XPCS) (Shpyrko, 2014 ▸; Narayanan *et al.*, 2023 ▸) and phase-contrast imaging (Xian *et al.*, 2022 ▸). At the same time, they introduce new challenges in beam characterization.

A key parameter in evaluating synchrotron performance is the electron source emittance, which directly affects the transverse coherence of the X-ray beam. The APS upgrade significantly reduces the natural emittance to a designed value of 42 pm rad. Measuring the source size with high precision is critical for validating the upgraded machine performance and confirming that the emittance values match the expected design parameters. Beyond machine verification, beamline operations require a detailed understanding of the transverse coherence function, as optimizing coherence-sensitive experiments depends on the precise characterization and understanding of how the beam propagates through the optical elements and experimental setups. Furthermore, minimizing coherence degradation caused by optical aberrations, misalignments or mechanical vibrations is necessary to maintain high experimental precision.

Several established techniques are available for measuring the source size of synchrotron beams (Samadi *et al.*, 2020 ▸; Samadi *et al.*, 2022 ▸). Pinhole imaging (Thomas *et al.*, 2010 ▸; Elleaume *et al.*, 1995 ▸) is widely used for direct source profile measurement, providing a spatially resolved source map, but suffers from diffraction effects, detector resolution limits and challenges in measuring ultra-small source sizes (Samadi *et al.*, 2020 ▸). Double-slit interferometry offers an alternative approach by measuring the transverse coherence length, but it has a limited dynamic range and requires careful alignment to extract reliable results (Samadi *et al.*, 2020 ▸). Near-field speckle-based methods have also been demonstrated for 2D source size and coherence measurements; however, they are not model-free and require accurate knowledge of the detector’s resolution function to yield reliable results (Kashyap *et al.*, 2015 ▸; Siano *et al.*, 2022 ▸). Grating interferometry (Marathe *et al.*, 2014 ▸; Shi *et al.*, 2014 ▸; Marathe *et al.*, 2016 ▸; Grizolli *et al.*, 2019 ▸) has emerged as a robust and quantitative method for coherence characterization, providing high sensitivity and direct access to the transverse coherence properties. Grating interferometry is model-free, allowing simultaneous extraction of source properties in both transverse directions, making it an attractive option for low-emittance sources. While this method has been successfully applied to the APS prior to its upgrade (Shi *et al.*, 2022 ▸), its application to the upgraded APS, where source sizes are significantly smaller, requires further refinements and validation.

This study provides an in-depth experimental analysis of the source emittance and beam coherence properties at the upgraded APS, using grating interferometry. Measurements were conducted at the APS 3-ID-B undulator beamline under two machine coupling conditions. The results confirm a world-record horizontal emittance of less than 30 pm rad. To further validate the methodology, we perform source size measurements at 1-BM-B (Macrander *et al.*, 2016 ▸), providing a baseline reference for a bending magnet source. Additionally, we investigate the effects of beamline optics on coherence preservation at the 12-ID-C beamline, analyzing the impact of optical aberrations and mechanical vibrations on the beam coherence. These measurements offer valuable insights into the upgraded APS performance and provide critical information for future beamline improvements. It also confirms the viability of grating interferometry as a precise tool for characterizing the X-ray source size and emittance of next-generation synchrotrons.

## 2. Grating interferometry for transverse coherence and source size measurements (Marathe *et al.*, 2014 ▸; Shi *et al.*, 2014 ▸; Marathe *et al.*, 2016 ▸; Grizolli *et al.*, 2019 ▸)

Transverse coherence measurement using grating interferometry exploits the van Cittert–Zernike theorem (Born & Wolf, 1999 ▸), which relates the coherence function at an observation plane to the Fourier transform of the source intensity distribution. In this method, a transmission grating placed in the X-ray beam path produces diffraction patterns that form periodic intensity fringes, or interferograms, at well defined distances downstream of the grating. The visibility of these interferograms, defined as the contrast of the intensity modulation, contains information about the transverse coherence of the beam. Since the coherence function is directly linked to the spatial extent of the source, analyzing visibility as a function of propagation distance enables precise determination of the apparent source size.

For a fully coherent source, the visibility of the interferograms remains constant regardless of the grating-to-detector distance *z*. In contrast, for a partially coherent source, the superposition of interferograms from the entire source opening angle (determined by the ratio of the source size to the source-to-grating distance) leads to a reduction in fringe contrast as *z* increases. This reduction in fringe contrast, quantified by the visibility decay as a function of *z*, directly determines the transverse coherence length and the apparent source size.

### 2.1. Experimental setup

**Figure 1:**
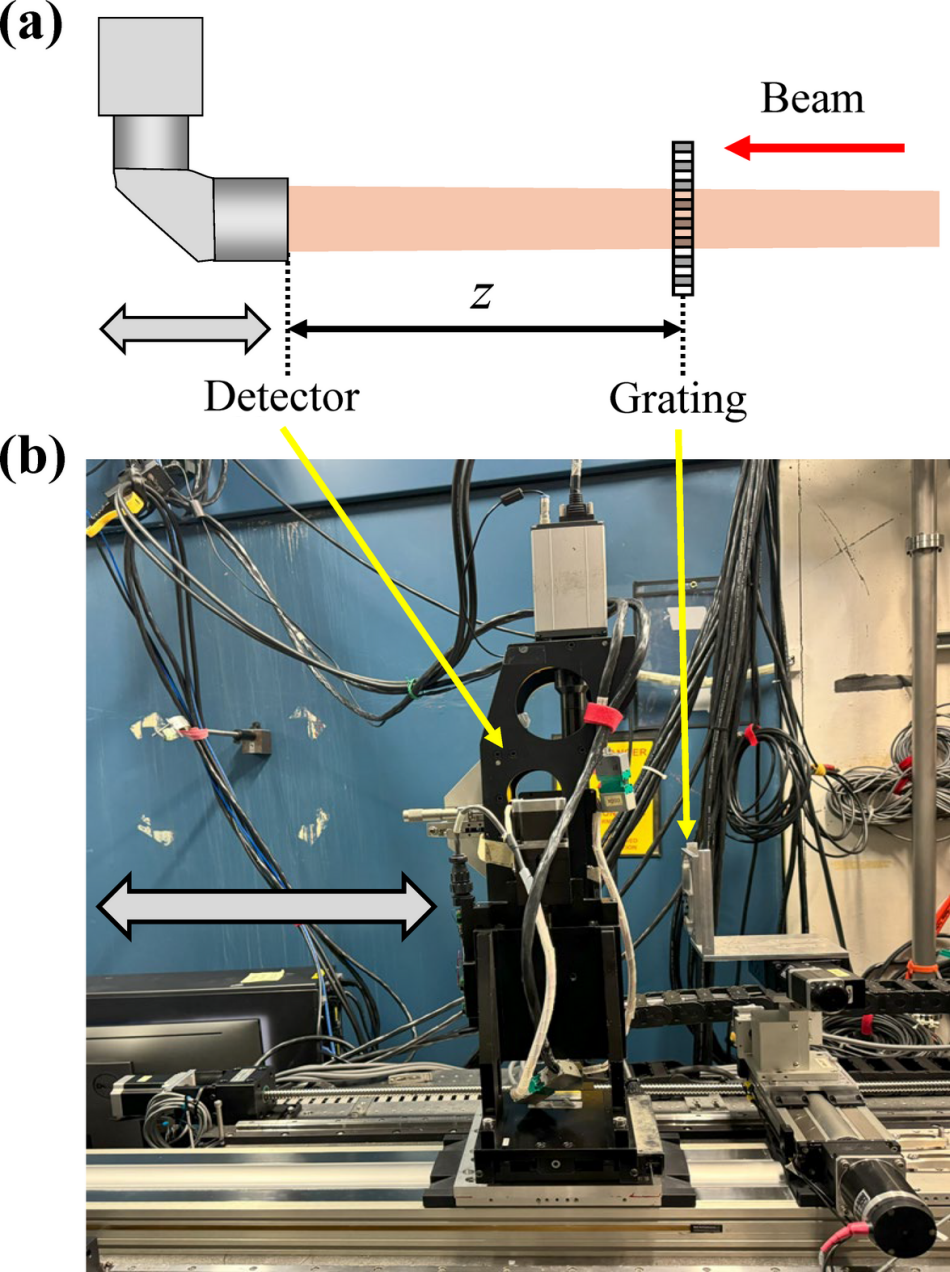
(*a*) Schematic of the grating interferometry setup. (*b*) Photograph of the experimental setup at the APS 3-ID-B beamline.

A schematic of the experimental configuration for grating interferometry (Assoufid *et al.*, 2016 ▸) is shown in Fig. 1 ▸(*a*), and a photograph of the actual setup at the APS 3-ID-B beamline is provided in Fig. 1 ▸(*b*). A transmission grating was positioned in the X-ray beam path at a fixed distance *D* from the source. Downstream of the grating, a scintillator-based detector system was mounted on a motorized stage, allowing precise control of the grating-to-detector distance *z*. Interferograms were recorded at multiple *z* positions to study the coherence properties of the beam. Additionally, the detector stage was equipped with *x*–*y* positioning capabilities, allowing the beam to be consistently aligned within the central region of the detector at each *z* position. This ensured consistent imaging conditions across all measurements, eliminating any ambiguity in image contrast caused by resolution differences across the detector surface.

The grating was fabricated at the Center for Nanoscale Materials by first patterning polymer molds using electron beam lithography, followed by Au electroplating to form the periodic structure (Marathe *et al.*, 2016 ▸). The grating used in this work was a 2D checkerboard π-phase grating, where the Au-coated regions introduced a π phase shift relative to the uncoated areas at a photon energy of 11.3 keV. The checkerboard grating had a period of *p*_G_ = 3.4 µm, producing mesh-patterned interferograms. The pattern period *p*_*z*_ of an interferogram at a distance *z* from the grating is given by (Grizolli *et al.*, 2019 ▸)
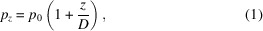
where *p*_0_ = *p*_G_/2 = 1.7 µm is the pattern period extrapolated to *z* = 0. To resolve the interferogram patterns, the detector pixel size must be smaller than *p*_0_/2, satisfying the Nyquist sampling criterion. In this work, the detector system consists of a Zyla sCMOS camera coupled with a 10× objective, providing a combined effective pixel of 0.65 µm. The grating area is 3 mm × 3 mm, while the detector’s field of view is approximately 1.4 mm × 1.7 mm. For the emittance measurements at 3-ID-B, the beam size at the measurement plane was smaller than the detector’s field of view, allowing the beam divergence to be measured as well. More generally, in interferometry-based methods, capturing the full beam profile at the detector is not required for source size measurements as long as the full opening angle of the source, as seen from the measurement plane, is not clipped or obstructed.

### 2.2. Data analysis and interpretation

The analysis involved two key steps: (i) determining the effective source position and (ii) extracting the effective source size from the visibility decay. The data analysis was performed using the Python package *wavepy2*, available in the GitHub repository (https://github.com/APS-XSD-OPT-Group/Wavepy2).

#### 2.2.1. Determination of the effective source position

**Figure 2:**
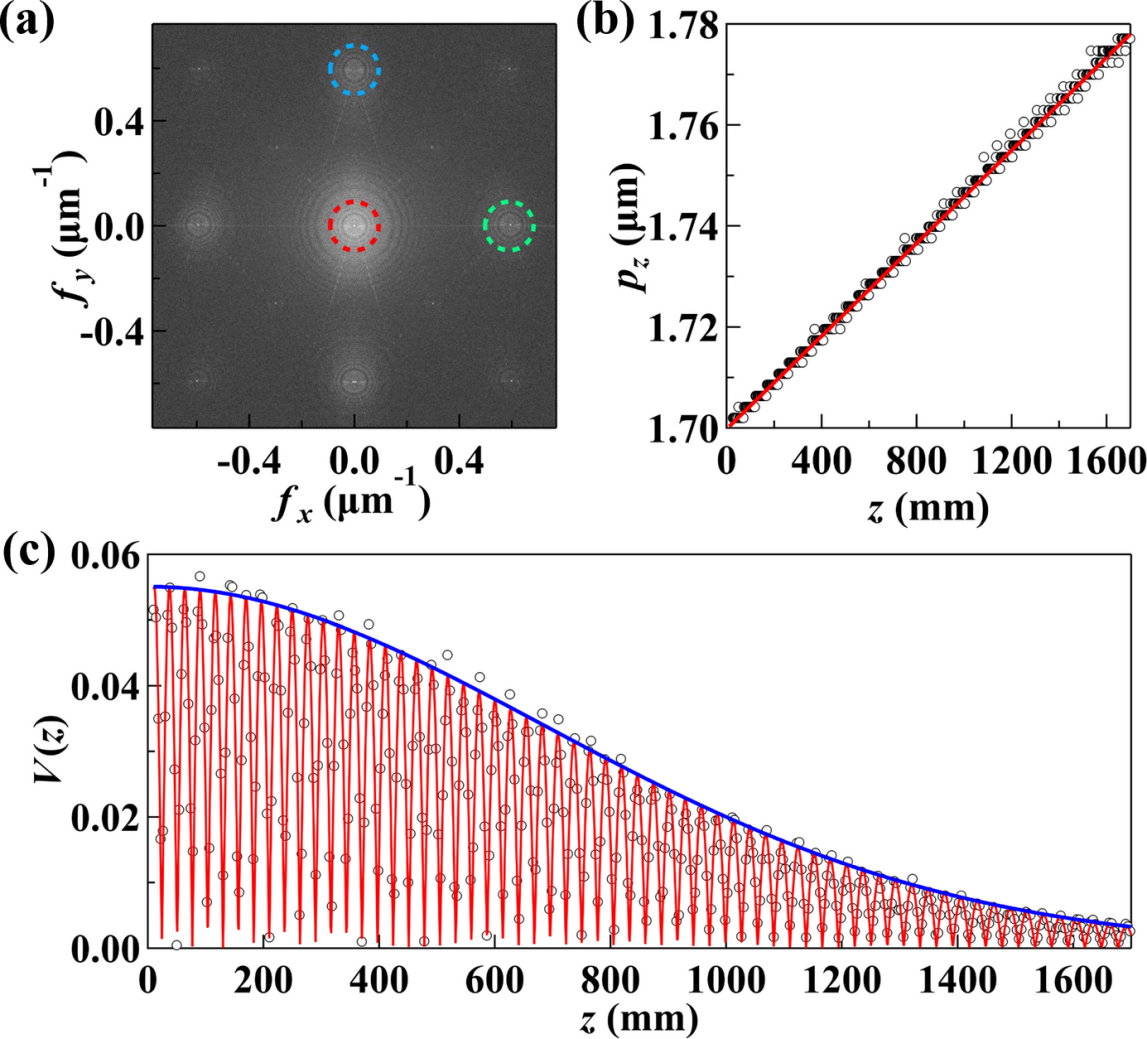
Representative examples of data analysis in grating interferometry, illustrating the key steps described in Sections 2.2.1 and 2.2.2. These examples are shown for demonstration purposes and are not necessarily linked to the beamline measurements presented later. (*a*) Fourier spectrum of an interferogram, highlighting harmonic peaks. (*b*) Interferogram period *p*_*z*_ as a function of *z*, with a linear fit. (*c*) Visibility curve *V*(*z*) with the red curve showing the full fit and the blue curve representing the coherence decay envelope.

At each detector position, corresponding to a grating-to-detector distance *z*, the interferogram period, *p*_*z*_, was obtained by identifying the first harmonic in the Fourier spectrum of the recorded interferogram. The first-harmonic frequency, denoted as *f*_*z*_, determines the interferogram period through the relation *p*_*z*_ = 1/*f*_*z*_. Fig. 2 ▸(*a*) shows the Fourier spectrum, where the first-harmonic peaks in the horizontal and vertical directions are marked by green and blue dotted circles, respectively.

Plotting *p*_*z*_ as a function of *z* [Fig. 2 ▸(*b*)] reveals a linear relationship consistent with the expected geometric expansion of a diverging beam originating from a source. A linear fit to these data yields two key parameters: the extrapolated period *p*_0_ at *z* = 0, and the source-to-grating distance *D*, determined from the slope *s* of the linear fit using the relation *D* = *p*_0_/*s*.

Since the 2D checkerboard grating generates interferograms with horizontal and vertical periodicity, the source position can be independently determined along each axis. This allows for direct assessment of beam astigmatism by comparing horizontal and vertical source positions, which can reveal optical imperfections or thermal loading effects in the beamline (discussed in Section 4). The analysis of the interferogram visibility decay, shown in Fig. 2 ▸(*c*), is presented next.

#### 2.2.2. Extraction of the effective source size from visibility decay

The second step in the analysis involved extracting the effective source size by analyzing the decay of the interferogram visibility *V*(*z*) as a function of *z*, as shown in Fig. 2 ▸(*c*). For a Gaussian distribution source with a root-mean-square (RMS) size Σ, the transverse coherence function at a distance *D* from the source is also a Gaussian profile, characterized by a coherence length ξ, given by (Onuki & Elleaume, 2002 ▸)
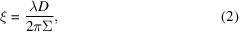
where λ is the X-ray wavelength.

Under these conditions, the interferogram visibility is described as the product of an oscillatory term due to the Talbot self-imaging effect and an exponential decay term, which accounts for partial coherence. The visibility function is expressed as (Grizolli *et al.*, 2019 ▸)
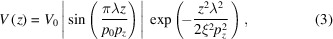
where *V*_0_  is a fitting parameter that accounts for additional blurring effects, such as detector resolution limits. The sine term captures the periodic contrast oscillation expected from the Talbot effect, with contrast peaks occurring at Talbot distances
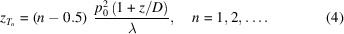
In the experiments, the visibility at each *z* was determined by computing the intensity ratio of the first-harmonic peak to the zero-harmonic component [the latter is marked by the red dotted circle in Fig. 2 ▸(*a*)]. By fitting the measured *V*(*z*) curve using equation (3), the coherence length ξ was extracted. Fig. 2 ▸(*c*) shows the fit, where the red curve represents the full visibility function, capturing both the oscillatory behavior and overall decay. The blue curve represents only the decay envelope from the exponential term in equation (3), which directly corresponds to the coherence function.

Once ξ is determined, the effective source size Σ is calculated using equation (2). The choice of *D* in these calculations is crucial. If *D* is taken from the *p*_*z*_ fitting in Fig. 2 ▸(*b*), it represents the effective source size at a virtual source point, which may not coincide with the electron beam waist. This information is valuable for determining downstream optics, such as adjusting the bending of a mirror to focus the virtual source at a desired sample plane. However, if the goal is to determine the electron source size at its waist, *D* should be set as the distance from the electron beam waist (*e.g.* the center of a straight section in the storage ring) to the measurement plane. In this case, the extracted effective size includes contributions from both the electron beam and the intrinsic photon emission. For example, an undulator source can be modeled as the convolution of the electron source profile with the photon emission distribution from a single electron.

A notable feature of this method is its ability to assess beam stability by varying the data acquisition time (Grizolli *et al.*, 2019 ▸; Shi *et al.*, 2022 ▸). If the acquisition time is much shorter than the vibration period, the measured source size reflects only the intrinsic source dimensions. Conversely, if the acquisition time spans multiple vibration cycles, the measured size includes motion-induced broadening, leading to a larger apparent source size. By comparing these two conditions, the contributions of vibrations and optical instabilities can be quantified over a frequency range defined by the two acquisition times. In cases where the acquisition time is approximately half of the vibration period, significant fluctuations in interferogram contrast can occur, as confirmed by statistical analysis of multiple measurements at each *z*.

## 3. Source size and emittance measurements at 3-ID-B and 1-BM-B beamlines

The transverse emittance of an electron beam is defined as the area it occupies in phase space. For a Gaussian beam, the geometric emittance ɛ is given by the product of the RMS source size σ and RMS divergence σ′ at the beam waist, ɛ = σσ′. Instead of directly measuring both size and divergence, an alternative approach is to measure the RMS source size σ and use the beta function derived from a calibrated lattice model. In either method, accurately measuring the electron source size at the waist remains the most challenging aspect.

Using the grating interferometry method, we first measured the source size at an undulator beamline. The APS 3-ID-B beamline is equipped with two inline U27 undulators, each with a 2.7 cm period and 87 periods. To minimize alignment effects between them, only the upstream undulator was used. Its gap was set to produce a first-harmonic resonance at 11.3 keV, where the flux is half of the spectral peak, and the undulator radiation closely follows a Gaussian profile (Onuki & Elleaume, 2002 ▸). The only optical element directly interacting with the X-ray beam was a vertically deflecting double-crystal monochromator tuned to 11.3 keV using the Si(111) reflection. To suppress third-order harmonic contamination at 33.9 keV, the relative angle between the two monochromator crystals was detuned, reducing the beam intensity by a factor of two. The electron source-to-grating distance was *D* = 36.5 m.

**Figure 3:**
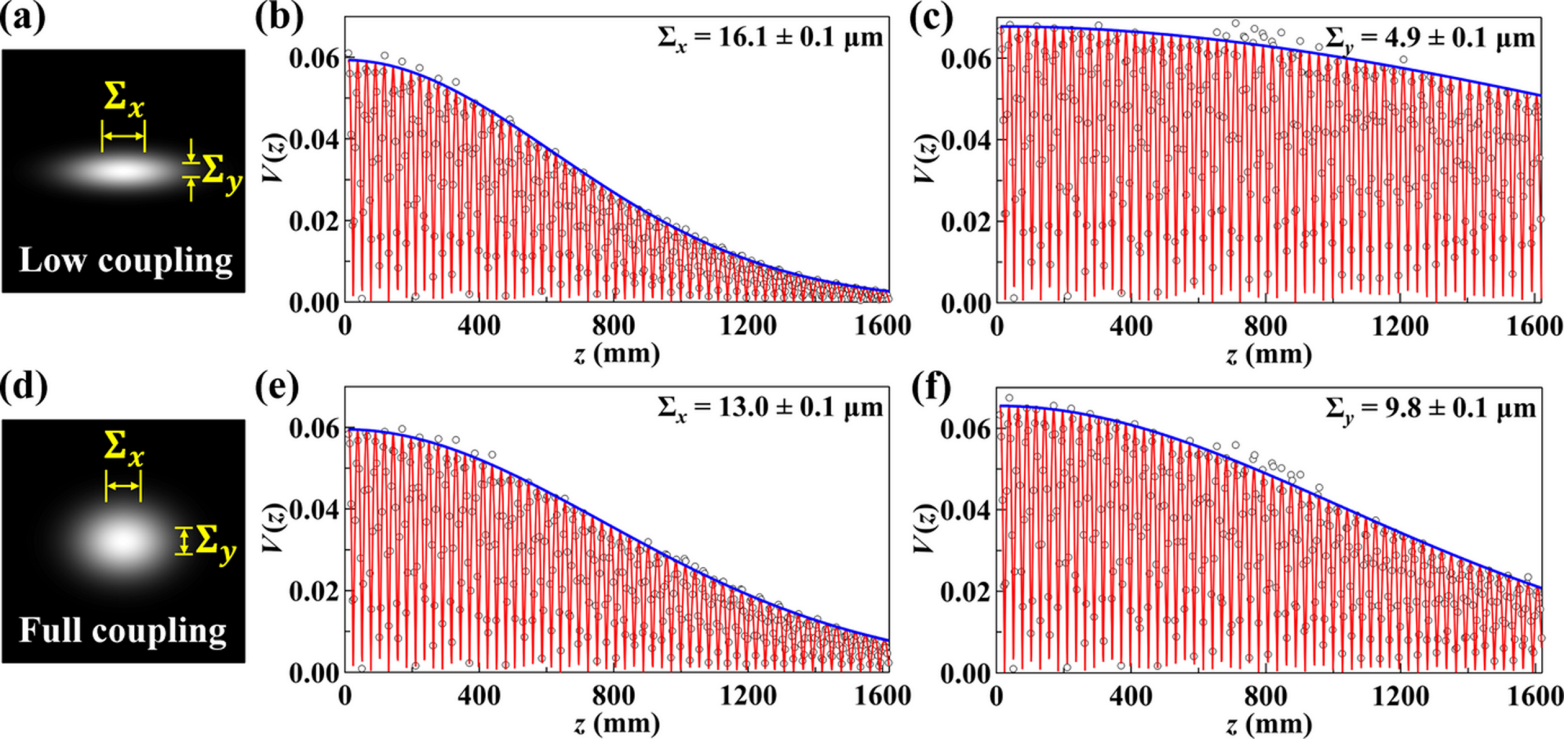
Source size measurements at the APS 3-ID-B beamline under two machine coupling conditions. (*a*) and (*d*) show schematics of the source shapes for low coupling and full coupling, respectively. These are Gaussian profiles generated using the measured sigma values to indicate the relative scale and shape of the source. (*b*, *c*, *e*, *f*) display the measured visibility *V*(*z*) as a function of *z* for the horizontal and vertical directions, with fitted curves using equation (3).

Source properties at 3-ID-B were measured under two different machine coupling conditions: low coupling, optimized for maximum brightness, and full coupling, which produces a nearly round source. Interferograms were acquired with a 20 ms exposure time, covering a grating-to-detector distance range from *z* = 10 mm to 1618 mm in 4 mm steps. The extracted visibility data and corresponding curve fits are presented in Fig. 3 ▸, along with the effective source sizes for both coupling conditions.

In the fitting of equation (3), the only free parameter was the coherence length ξ [or source size Σ through equation (2)], while *p*_0_ was determined from the *p*_*z*_ fitting using equation (1). The fitted distance *D* agrees with the expected source location within an uncertainty of less than 0.5 m, and a fixed value of *D* = 36.5 m was used consistently for source size calculations. The excellent agreement between the measured visibility and the fitted curves highlights the robustness of the method, which benefits from significant oversampling compared with traditional single-shot techniques such as pinhole imaging. With hundreds of interferograms contributing to a fit involving only a few free parameters, the method effectively mitigates the impact of single-measurement errors, including detector noise, limited resolution, beam instabilities, environmental fluctuations and artifacts from non-ideal fringe patterns in the Fourier analysis. This approach enhances the overall robustness of the measurements, ensuring that occasional outliers have minimal influence on the extracted source parameters.

The experimentally determined total source size Σ served as the starting point for analysis. The single-electron photon source size σ_ph_ was calculated using *Synchrotron Radiation Workshop* (*SRW*) (Chubar & Elleaume, 1998 ▸), and the electron source size σ_e_ was obtained by quadrature subtraction of the photon source size from the total source size,
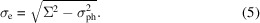
To calculate the emittance, beam divergence information was also required. The total beam divergence Σ′ was experimentally determined by measuring the full beam size σ_beam_ at various distances downstream of the source, corresponding to detector positions at *D* + *z*. These measurements were performed using the same detector system, with the grating removed from the beam path. The divergence Σ′ was extracted as the slope of a linear fit to σ_beam_ as a function of *D* + *z*. Similarly, the single-electron photon divergence σ_ph_′ was calculated using *SRW* simulations, and the electron beam divergence σ_e_′ was determined through quadrature subtraction,
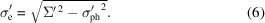
The emittance in horizontal and vertical directions was then obtained for the two coupling conditions (full coupling and low coupling), with the results summarized in Table 1 ▸. The measurement uncertainty in the total source size Σ is ±0.1 µm, determined from the model fitting of the visibility curve. The uncertainties in beam divergence Σ′ are ±0.1 µrad and ±0.03 µrad for the horizontal and vertical directions, respectively, obtained from the variation in beam size measured at a fixed distance over 100 images. The uncertainties in the simulated single-electron photon source size and divergence, calculated using *SRW*, are challenging to determine precisely. However, using values of ±0.1 µm for σ_ph_ and ±0.1 µrad for σ_ph_′, similar to the measurement uncertainties, provides a reasonable estimate for the emittance error analysis. The final uncertainty in emittance was calculated by standard error propagation through ɛ = σ_e_σ_e_′ incorporating the relationships defined in equation (5) and equation (6).

Taken together with the uncertainty analysis, the experimentally determined emittances in Table 1 ▸ strongly agree with the emittance values derived from the measured storage ring model. Theoretical betatron emittances for the full coupling and zero coupling conditions are also included for reference. These results not only confirm the designed emittance values of the upgraded APS source but also demonstrate the world-record horizontal emittance below 30 pm rad, underscoring the success of the APS upgrade.

**Figure 4:**
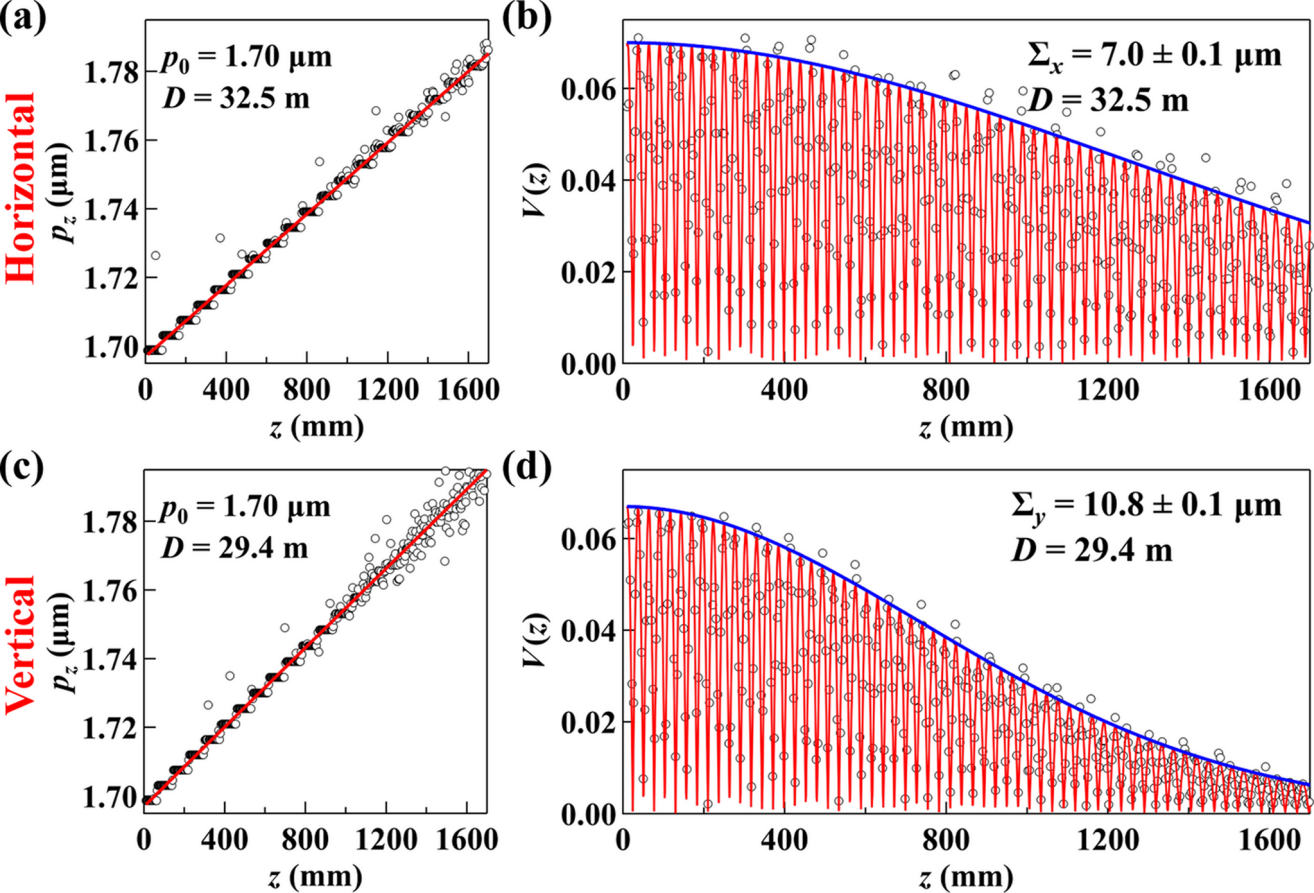
Source size measurements at the APS 1-BM-B beamline. (*a*) and (*c*) show the interferogram periods *p*_*z*_ as a function of *z* with linear fits, while (*b*) and (*d*) present the visibility curves *V*(*z*) with fits based on equation (3). Acquisition time for each interferogram is 100 ms.

Source size measurements were conducted at the APS 1-BM-B beamline using the same grating to further verify the source properties in the full coupling mode. At a bending magnet beamline, where the photon size contribution is negligible, grating interferometry provides a direct measurement of the electron source size. The monochromator setup was identical to that of 3-ID-B, with a vertically deflecting double-crystal Si(111) monochromator tuned to 11.3 keV and detuned to suppress third-order harmonic contamination. The electron source-to-grating distance was *D* = 32.5 m. The results are shown in Fig. 4 ▸.

In the horizontal plane, the measured source location aligns well with the physical source-to-grating distance at *D* = 32.5 m, yielding a horizontal electron source size of 7.0 µm, which closely agrees with the theoretical value of 6.9 µm predicted by machine modeling. In contrast, the vertical measurements reveal a slightly shorter effective source distance *D*_eff_, attributed to the thermal bump on the vertically deflecting monochromator. The effective vertical source size is measured as Σ_*y*,eff_ = 10.8 µm at a virtual source waist corresponding to *D*_eff_ = 29.4 m. Assuming the thermal bump primarily introduces a second-order effect, the monochromator behaves like an ideal convex mirror. Under this assumption, the source size at the physical electron waist at *D* = 32.5 m can be estimated by scaling, yielding Σ_*y*,eff_*D*/*D*_eff_ = 11.9 µm, which is in good agreement with the modeled value of 11.8 µm.

The electron source divergence cannot be directly measured at a bending magnet beamline due to the dominance of photon divergence. However, using the measured source sizes and the modeled beta function, the calculated emittances are 35 pm rad in the horizontal direction and 31 pm rad in the vertical direction. These values are consistent with the predicted emittances of 34 pm rad and 31 pm rad, further validating the machine parameters. It should be noted that the 1-BM-B measurement was conducted at a beam current of 180 mA on a different day than the 3-ID-B measurement, leading to slight differences in the emittance values.

Both beamlines were selected for emittance measurements due to their simple optical layouts, each containing only a single vertically deflecting monochromator. At 3-ID-B, measurements conducted at a 50 mA electron beam current showed minimal thermal effects, with the extracted vertical source position matching the physical source, indicating negligible distortion. In contrast, measurements at 1-BM-B, taken at 180 mA, revealed a vertical source position shift attributed to thermal deformation. These observations provide context for the coherence preservation analysis discussed next.

## 4. Effects of beamline optics on coherence properties

Although source emittance is a fundamental machine parameter, transverse beam coherence is more directly relevant to beamline operations and the design of coherence-based experiments. At the sample position, the beam results not only from the pristine source but also from the cumulative influence of all intervening optical elements. This section examines how optical instabilities and aberrations affect beam coherence, characterized by the effective source size.

Measurements at the APS 12-ID-C beamline provide a unique opportunity to study these effects, serving as a baseline with legacy optics while benefiting from the upgraded source. By applying grating interferometry, we can isolate and quantify the contributions of mechanical vibrations and optical imperfections to the effective source size. These measurements offer detailed insights into the current beamline performance and serve as a benchmark for the planned refurbishment of legacy optics. The results underscore the effectiveness of grating interferometry in diagnosing coherence degradation and guiding improvements in beamline performance.

The measurements were performed under the full coupling machine mode. We used the upstream 2.1 m-long undulator with a 2.8 cm period and 74 periods. A vertical deflecting double-crystal monochromator, located 33.0 m from the source, was used to tune the X-ray energy to 10.8 keV. A horizontal mirror, located 28.7 m from the source, is equipped with back-heaters that allow controlled bending to adjust the horizontal beam divergence. The measurements were carried out at a distance of *D* = 59.5 m from the source.

### 4.1. Instability effects on beam coherence

**Figure 5:**
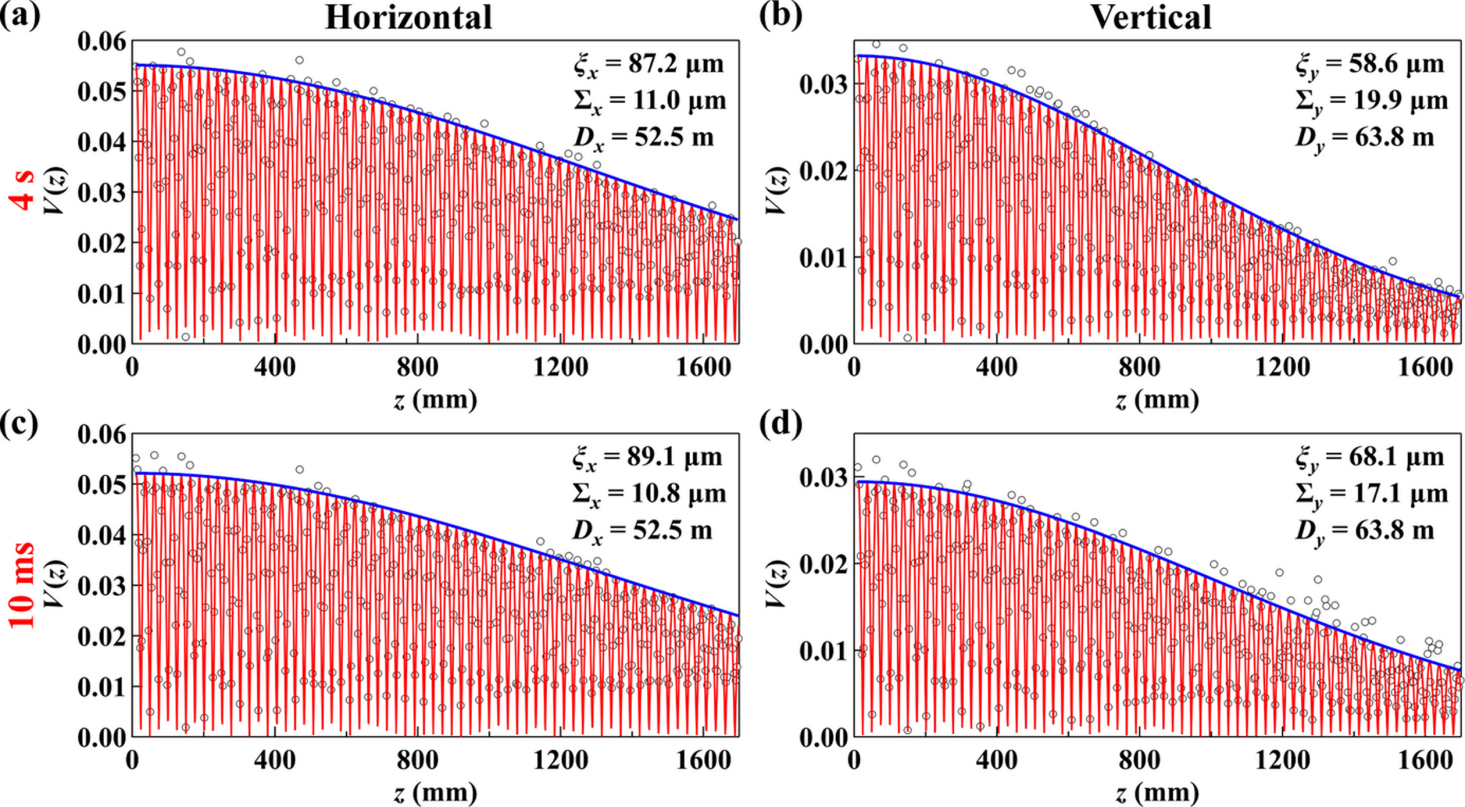
Source size measurements at APS 12-ID-C with different acquisition times: (*a*) horizontal 4 s; (*b*) vertical 4 s; (*c*) horizontal 10 ms; (*d*) vertical 10 ms.

To investigate the impact of optical instabilities on beam coherence, we measured the transverse coherence using two different acquisition times: 10 ms and 4 s. The extracted coherence length and the effective source sizes at their apparent distances are compared in Fig. 5 ▸. In the horizontal direction, the coherence lengths (ξ_*x*,4s_ = 87.4 µm and ξ_*x*,10ms_ = 89.1 µm) and effective source sizes (Σ_*x*,4s_ = 11.0 µm and Σ_*x*,10ms_ = 10.8 µm) vary by less than 2%, indicating minimal vibrations within the 10 ms to 4 s frequency range. In contrast, the vertical coherence lengths (ξ_*y*,4s_ = 58.6 µm and ξ_*y*,10ms_ = 68.1 µm) and effective source sizes (Σ_*y*,4s_ = 19.9 µm and Σ_*y*,10ms_ = 17.1 µm) differ by 16%, suggesting more significant vibrations in that direction. Additionally, as seen in Fig. 5 ▸, the extracted virtual source positions deviate from the physical source-to-grating distance of 59.5 m. In the horizontal direction, the source appears at 52.5 m, corresponding to a 7.0 m downstream shift, indicating horizontal defocusing likely caused by aberrations from the horizontal mirror. In the vertical direction, the effective source position is measured at 63.8 m, or 4.3 m upstream of the physical source, likely due to the thermal deformation of the vertically deflecting monochromator. The impact of the horizontal mirror on beam coherence will be examined in more detail in Section 4.2.

**Figure 6:**
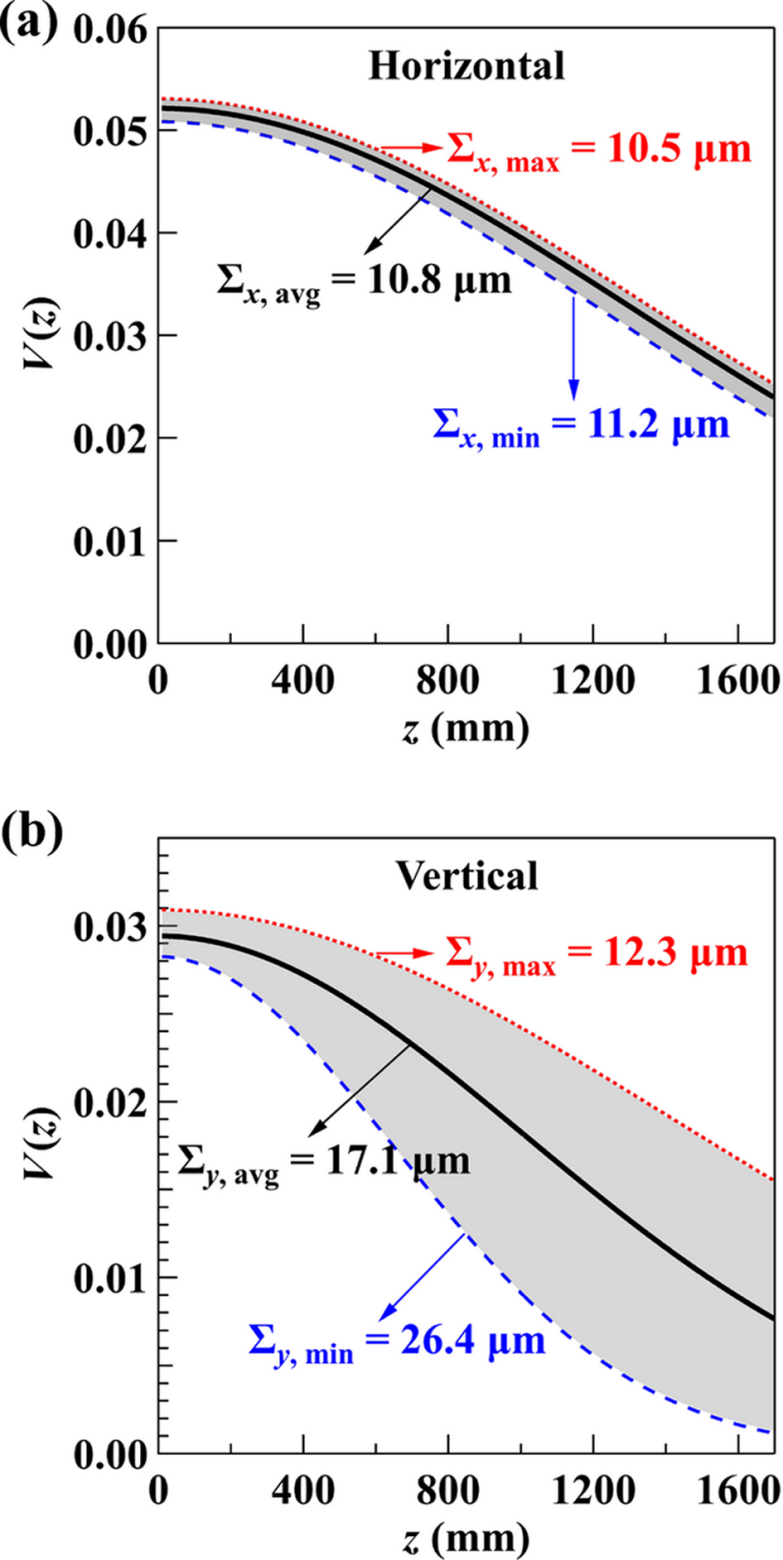
Visibility statistics from ten measurements with a 10 ms acquisition time as a function of *z* in the (*a*) horizontal and (*b*) vertical directions. The solid curves represent fits using the average visibility, while the dotted and dashed curves correspond to fits using the maximum and minimum visibilities, respectively, defining the shaded uncertainty range.

To gain further insight into beam stability, we acquired ten interferograms at each *z* position, with an acquisition time of 10 ms per image, and analyzed the statistical variations in their visibilities. The visibility curve was fitted using the maximum, average and minimum visibility of the ten images at each *z*. Fig. 6 ▸ shows these three fitted envelope functions (coherence function) in the horizontal and vertical directions, along with the corresponding source size values.

In the horizontal direction [Fig. 6 ▸(*a*)], the extracted source sizes show minimal deviation between the maximum, average and minimum visibility fits, confirming low horizontal vibration. In contrast, the vertical results in Fig. 6 ▸(*b*) show substantial deviations between the three curves, a characteristic signature of measurements taken at a frequency close to half of the vibration frequency (Grizolli *et al.*, 2019 ▸).

For a source oscillating transversely as a simple sinusoidal function of time, its motion speed varies depending on its position. The velocity is highest at the center of the oscillation range and lowest at the extremes. If measurements are taken when the source moves fastest (near the center), the broadening effect is maximized, as seen in the fit using the minimum visibility at each *z* with Σ_*y*,min,10ms_ = 26.4 µm. Conversely, when the source is near its slowest motion, the measured source size approaches its static value without vibration, as indicated by Σ_*y*,max,10ms_ = 12.3 µm.

The result from the long acquisition time [Σ_*y*,4s_ = 19.9 µm, shown in Fig. 5 ▸(*b*)] indicates more than 60% broadening compared with Σ_*y*,max,10ms_, reflecting the influence of vertical vibrations occurring on timescales between 10 ms and 4 s. This observed vertical instability arises from a combination of the electron source motion and mechanical vibrations of the monochromator. Grating interferometry is primarily sensitive to the positional, rather than angular, motion of the electron source, making it well suited for isolating these effects. Since vertical fluctuations in the electron source position are less than 10% of the source size, their broadening to the source size is negligible (less than 1% in quadrature). Therefore, the dominant source of vertical size broadening is attributed to monochromator vibrations, primarily the relative angular motion between the two crystals, which induces oscillations in the virtual source position. Considering the monochromator is located 33.0 m downstream from the physical electron source, and the effective vertical source position was measured to be 4.3 m upstream of that physical source point, the total lever arm becomes *D*_*M*_ = 37.3 m. The RMS angular vibration of the monochromator is then estimated as
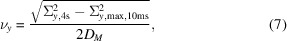
which yields ν_*y*_ = 0.21 µrad, a value commonly observed at third-generation synchrotrons. For comparison, the APS upgrade specifies new monochromators with an expected angular stability better than 0.05 µrad, which would broaden the source size by less than 5%. While this method estimates coherence degradation over a defined timescale, obtaining the vibration frequency spectrum would require complementary techniques, such as power spectral density (PSD) analysis of partially blocked beam intensity fluctuations monitored by a fast detector (*e.g.* PIN diode).

### 4.2. Optics aberration effects on beam coherence

From Fig. 5 ▸, we determined that the horizontal virtual source is located 52.5 m upstream of the measurement position. This virtual source position is 7.0 m downstream from the electron source waist. The only optical element affecting the beam in the horizontal direction is the horizontally reflecting white-beam mirror. The results suggest this mirror has a convex surface shape, likely due to beam thermal loading. This is a typical example of how the local surface curvature of reflective optics, such as mirrors, impacts the beam coherence and effective source.

**Figure 7:**
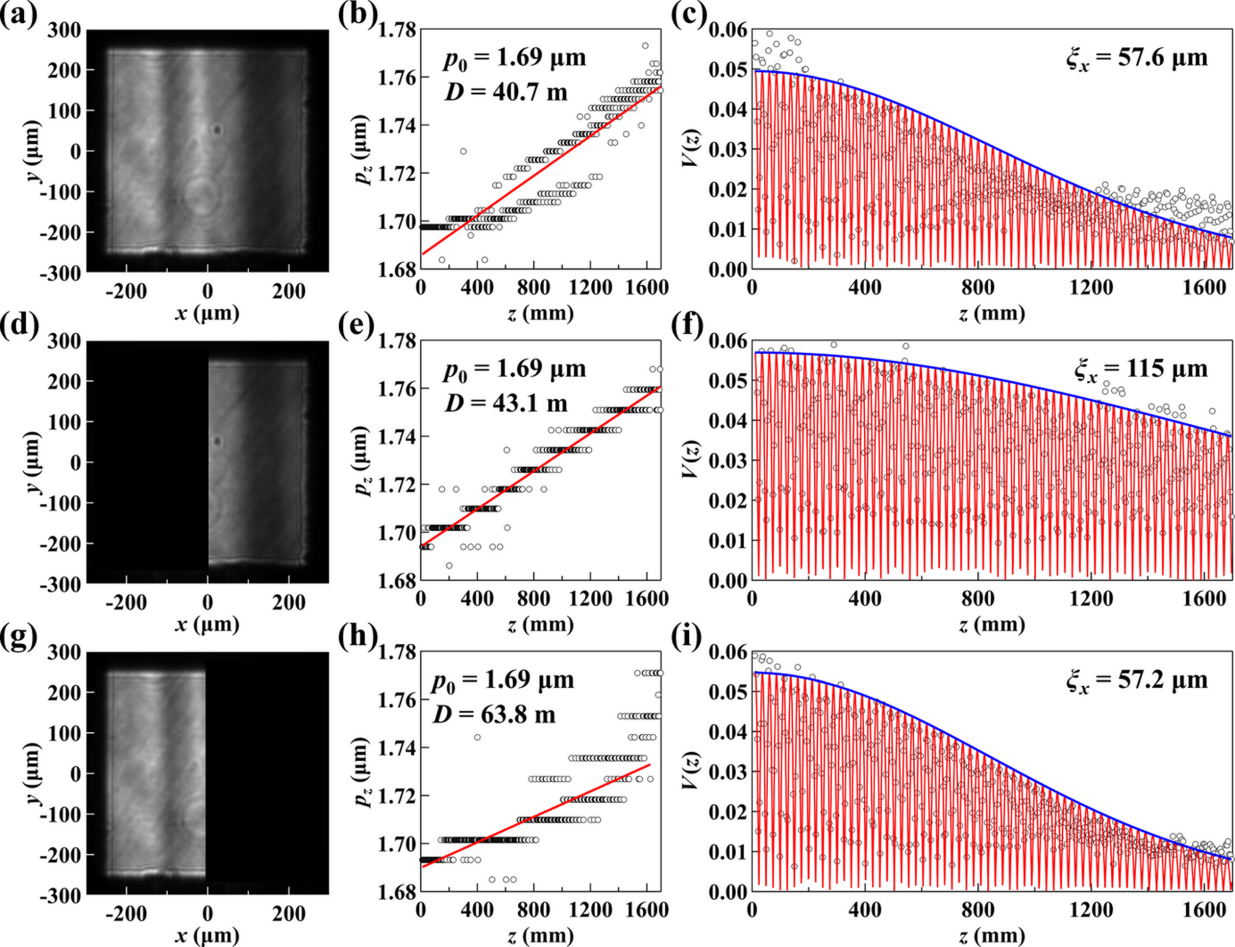
Grating interferometry measurements with different horizontal analysis apertures. (*a*), (*d*) and (*g*) show the beam within different selected analysis apertures. (*b*), (*e*) and (*h*) display the interferogram periods *p*_*z*_ as a function of *z*, with linear fits used to determine the effective source position *D*. (*c*), (*f*) and (*i*) present the visibility curves *V*(*z*) with fits based on equation (3), revealing the transverse coherence length at the measurement plane.

To investigate this effect, a power of 9 W was applied uniformly to all six heating electrodes attached to the back of the mirror. This value was chosen arbitrarily to induce measurable thermal gradients rather than to reflect an optimized operating condition. The resulting thermal gradients introduced local curvatures on the mirror surface, modifying the beam’s horizontal wavefront curvature. Measurements were then performed under these conditions and analyzed using different horizontal analysis apertures. The results are summarized in Fig. 7 ▸.

For the full aperture measurement, as shown in Figs. 7 ▸(*a*)–7 ▸(*c*), the results exhibit poor fitting in both the *p*_*z*_ and *V*(*z*) curves. This is due to the presence of multiple apparent sources defined by local wavefront curvatures. For example, the right (inboard) half of the beam displays a distinct cylindrical wavefront shape [Fig. 7 ▸(*d*)], which corresponds to a virtual source position located at 43.1 m upstream of the measurement position, as shown in Fig. 7 ▸(*e*). This portion of the beam has a coherence length of ξ_*x*_ = 115 µm [Fig. 7 ▸(*f*)]. In contrast, the left (outboard) half of the beam has an effective source much further upstream at 63.8 m [Fig. 7 ▸(*h*)], indicating a less divergent beam with a coherence length of ξ_*x*_ = 57.2 µm [Fig. 7 ▸(*i*)]. This observation is consistent with the higher average intensity observed on the left side, which appears brighter in the grayscale image of Fig. 7 ▸(*g*) compared with Fig. 7 ▸(*d*).

When using a beam with significant wavefront curvature variations, careful consideration must be given to its application. For experiments requiring only a tiny portion of the beam, selecting a region that matches the required coherence can be advantageous. Conversely, experiments that utilize the entire beam will experience reduced overall coherence due to the combined contributions from multiple effective sources located at different positions.

## 5. Conclusion

This study presents a comprehensive characterization of the upgraded APS source emittance and beam coherence properties using grating interferometry. The results confirm the design parameters of the upgraded APS source, demonstrating a world-record horizontal emittance. Source size measurements at both undulator and bending magnet beamlines verify the low-emittance source, showing strong agreement between experimental values and theoretical predictions.

Grating interferometry measurements at another undulator beamline highlight the impact of legacy optics on coherence preservation. Optical aberrations, such as thermal-induced mirror curvature, significantly affect the effective source size, while mechanical vibrations contribute to coherence degradation. These findings emphasize the importance of high-quality optics, precise alignment and mechanical stability in maintaining beam coherence.

Future work will extend coherence studies to additional beamlines and integrate advanced wavefront sensing techniques for beam quality evaluation. These efforts will establish a baseline for optimizing beamline optics, ensuring full utilization of the upgraded APS source. In synergy with ongoing developments, including AI- and machine-learning-driven auto-alignment systems (Rebuffi *et al.*, 2023*a* ▸; Rebuffi *et al.*, 2023*b* ▸), as well as adaptive and corrective optics to compensate for existing imperfections, these advancements will collectively enhance beam stability, improve coherence preservation, and enable next-generation synchrotron applications and scientific discoveries.

## Figures and Tables

**Table 1:** Summary of measured and derived source parameters at the APS 3-ID-B beamline with X-rays at 11.3 keV; measurements were performed at a beam current of 50 mA under different machine coupling conditions

	Full coupling		Low coupling	
	Horizontal	Vertical	Horizontal	Vertical
Total source size, Σ (µm)	13.0	9.8	16.1	4.9
Single-electron photon size, σ_ph_ (µm)	4.0	4.0	4.0	4.0
Electron source size, σ_e_ (µm)	12.4	8.9	15.6	2.8
Total beam divergence, Σ′ (µrad)	4.9	5.6	5.2	4.5
Single-electron photon divergence, σ_ph_′ (µrad)	4.4	4.4	4.4	4.4
Electron beam divergence, σ_e_′ (µrad)	2.2	3.5	2.8	1.2
Measured emittance, ɛ = σ_e_σ_e_′ (pm rad)	28 ± 3	31 ± 2	44 ± 3	3.3 ± 2
Emittance from storage ring model† (pm rad)	31	31	44	2.9
Theoretical betatron emittance (pm rad)	29	29	42‡	0‡

† Model is derived from the response matrix fit.

‡ The value corresponds to the theoretical zero-coupling condition.

## Data Availability

Data presented in this paper are not publicly available but may be obtained from the authors upon request.
